# High Molecular Weight Typing with MALDI-TOF MS - A Novel Method for Rapid Typing of *Clostridium difficile*

**DOI:** 10.1371/journal.pone.0122457

**Published:** 2015-04-29

**Authors:** Kristina Rizzardi, Thomas Åkerlund

**Affiliations:** Department of Microbiology, Public Health Agency of Sweden, Solna, Sweden; Institute Pasteur, FRANCE

## Abstract

*Clostridium difficile* strains were typed by a newly developed MALDI-TOF method, high molecular weight typing, and compared to PCR ribotyping. Among 500 isolates representing 59 PCR ribotypes a total of 35 high molecular weight types could be resolved. Although less discriminatory than PCR ribotyping, the method is extremely fast and simple, and supports for cost-effective screening of isolates during outbreak situations.

## Introduction

*Clostridium difficile* is a common cause of diarrhea mainly in elderly hospitalized patients and, other than age, antibiotic treatment and the length of hospital stay are well-known risk factors [1]. A number of strain types have been identified as potential outbreak-related and molecular typing, e.g. PCR ribotyping, PFGE and MLVA, has become an important tool to identify such outbreaks [2]. MALDI-TOF MS has recently become widely used for microorganism identification, mainly because the technique is fast, cheap and reliable [3,4]. MALDI-TOF MS generates protein fingerprints of whole cells, these are used to identify microorganisms by matching against a database. Unique protein peaks within a spectrum can also be used as biomarkers to differentiate between subspecies or even for typing. Its potential also for subtyping of *C*. *difficile* was noted by Reil *et al*. [5] where MALDI-TOF MS was tested on 29 PCR ribotypes of *C*. *difficile* using peaks in the 2–20 kDa range. However, the published data could only discriminate between the PCR ribotypes 001, 027 and 078/126. In this study we developed a MALDI-TOF method based on the high molecular weight (HMW) protein profile (mass range of 30-50kDa), from whole cells, followed by analysis of peaks in the 2–20 kDa range from protein extractions, and evaluated the results against PCR ribotyping as reference method.

## Material and Methods

### Bacterial strains and culture conditions

An initial MALDI-TOF database was constructed using the Cardiff-ECDC reference strains (PCR ribotypes 001, 002, 003, 012, 014/077, 015, 017, 020, 023, 027, 029, 046, 053, 056, 078/126, 081, 087, 095, 106, 117) and 10 clinical isolates from each of the 15 most common PCR-ribotypes in Sweden (001, 002, 003, 005, 012, 014/077, 017, 020, 023, 029, 046, 078, 081, 220, 231). Validation was performed using 222 isolates collected from 26 clinical laboratories in Sweden, typed by PCR ribotyping in parallel (below). The high molecular weight profiles of PCR ribotypes not included during the initial method setup but detected in the validation cohort were confirmed by analyzing 108 additional isolates from our biobank. In total, 500 isolates were analyzed in triplicate by MALDI-TOF. No difference was observed between runs in regard to technical replicates. All isolates were grown for 48h at 35°C on blood agar plates under anaerobic conditions.

### S-layer protein (SLP) extraction

SLPs were extracted by two different methods, an 8M urea extraction described by Cerquetti *et al*. [6], and a 0.2M acidic glycine extraction described by Calabi *et al*. [7]. After extraction the SLP fraction was concentrated and desalted on Centri-Sep spin columns (Life Technologies) prior to MALDI-TOF MS analysis. 1 μl desalted SLP extract was analyzed by MALDI-TOF MS as described below.

### MALDI-TOF sample preparation

HMW-profiles were detected with whole cell mass spectrometry (WCMS), single colonies were directly transferred to a spot on the MALDI target plate and overlaid with 1 μl trans-ferulic acid matrix solution [8] (FerA, Sigma-aldrich; 15mg/ml of FerA dissolved in 33% acetonitrile, 17% formic acid (FA) and 50% H_2_O). Ethanol/formic acid (EtOH/FA) extraction and direct transfer followed by application of 1 μl FA was performed following Brukers’ standard protocol using α-cyano-4-hydroxycinnamic acid matrix (HCCA, Bruker) [9].

### MALDI-TOF MS parameters and spectral analysis

All spectra were collected on a microflex LT using flexControl (Bruker). A new method was generated, for analysis using FerA matrix; running in linear positive mode with a laser frequency of 60 Hz (laser power 90% to 100%, offset 15%, range 30%), delayed ion extraction of 100 ns, acceleration voltage and lens voltage of 20 kV and 6 kV respectively and a mass range of 2000 to 80000 m/z. For automatic sample collection an AutoXecute method was generated, peak selection uses masses from 6000 to 50000 m/z for evaluation without ignoring the largest peak, 240 shots in 40 steps are collected for the final spectrum. A centroid peak detection algorithm is used with S/N ratio 5, peak width 40 m/z, height 90% and minimum intensity threshold 400. The FerA method was calibrated using a high molecular weight standard (Protein standard II, Bruker).

The standard Biotyper method (MBT_FC) was adopted for analysis using HCCA matrix, the method was calibrated using a bacterial test standard (BTS, Bruker). All spectra were analyzed with the Flex Analysis software and processed with smoothening and baseline subtraction (MBT_standard.FAMSmethod). Peak selection for the FerA spectra was performed in Flex Analysis using a centroid algorithm, S/N ratio 2, peak width 50 m/z, minimum intensity threshold 200, top hat baseline subtraction and Savitzky/Golay smoothing. Spectra from EtOH/FA extractions were aligned using internal recalibration, *C*. *difficile* peak 5435.50 m/z, mass calculated using BTS mixed with *C*. *difficile* EtOH/FA extract. Peak selection for the HCCA spectra was performed in Flex Analysis using a centroid algorithm, S/N ratio 5, peak width 0.2 m/z minimum intensity threshold 250, top hat baseline subtraction and Savitzky/Golay smoothing.

### PCR ribotyping

A national PCR ribotype database based on capillary gel electrophoresis was constructed, containing up to date a total of 1179 *C*. *difficile*-isolates collected in the Swedish national surveillance program during 2009–2012 [10]. PCR ribotyping was a modification of the method by Indra *et al*. [11]. For template preparation, 50 μl culture (PY-cultures grown for 18–20 h) were frozen in aliquots, thawed and diluted in 150 μl 10% Chelex-100 (Biorad) in water. The solution was vortexed, heated in 95 degrees for 10 min, centrifuged, and 10 μl was diluted in 90 μl water (final template). 2.2 μl template was added to 2.8 μl PCR mix containing 2.5 μl AmpliTaq Gold Fast PCR master mix and 0.3 μl of primers (FAM-labeled 16S-primer 5’-GTG CGG CTG GAT CAC CTC CT-3’ and 23S-primer 5’-CCC TGC ACC CTT AAT AAC TTG ACC-3’; final concentrations 0.04 μM and 0.24 μM, respectively). PCR was run in a Veriti Thermal Cycler (Applied Biosystems) using 96-well FAST plates and with the following program: 95°C for 10 minutes, 28 cycles of 96°C for 3 seconds, 65°C for 3 seconds and 68°C for 15 seconds, followed by a final elongation of 72°C for 30 minutes. After run, each PCR product was diluted 1:1 in water.

### Capillary gel electrophoresis

Prior to PCR fragment analysis, GeneScan 1200 LIZ Size Standard was diluted 1:25 in Hi-Di Formamide. The capillary gel electrophoresis was performed on an ABI 3500xl Genetic Analyzer, with 50 cm capillaries (n = 24) loaded with POP7, in 10 μl reactions with 9.5 μl LIZ standard mix and 0.5 μl PCR-product. All reagents were from Applied Biosystems.

### Data analysis

Capillary gel electrophoresis data files were imported into Bionumerics. Normalization was performed using the following Auto settings: Threshold and Valley depth = 2; Global alignment: Allowed expansion/compression = 2, Allowed displacement = 1; Local alignment: Allowed deformation = 15, Peak comparison location/shape = 0/100%. Band detection was performed in three steps: 1) Min profiling = 2, Gray zone = 0, Min area = 4, shoulder sensitivity = 1 and metrics range 200–350. 2) Min profiling = 2, Gray zone = 0, Min area = 1, shoulder sensitivity = 1 and metrics range 351–450 (keep existing bands). 3) Min profiling = 2, Gray zone = 0, Min area = 0.25, shoulder sensitivity = 1 and metrics range 451–620 (keep existing bands). Cluster analysis was performed using the following settings: Band based = Jaccard, Optimization = 0.1%, Tolerance and Tolerance change = 0.4%, Relaxed doublet matching and UPGMA. Isolates that did not match known PCR ribotypes were categorized as a new PCR ribotype with the prefix SE.

## Results and Discussion

The MALDI-TOF method was initially developed by empirically determine a suitable mass range to discriminate strains of the ECDC Cardiff collection and the most common PCR ribotypes in Sweden. Peaks were detected in the 30 to 50 kDa region which corresponds to the same size as the *C*. *difficile* S-layer proteins (SLPs). To verify that the major peaks corresponded to the SLPs, we performed an SLP extraction from strains of the ECDC Cardiff collection using two different methods [6, 7] and compared the profiles to those of the WCMS analysis by MALDI-TOF MS. A representative result of two PCR ribotypes (003 and 015) showed that the major peaks derived from the extraction procedures and the WCMS were of the same size, confirming that these indeed corresponded to the SLPs (S1 Fig). However, there were also additional peaks in the WCMS analysis that were missing from the SLP extraction spectra, suggesting that both the SLPs and other high molecular weight proteins are detected by WCMS.

**Fig 1:**
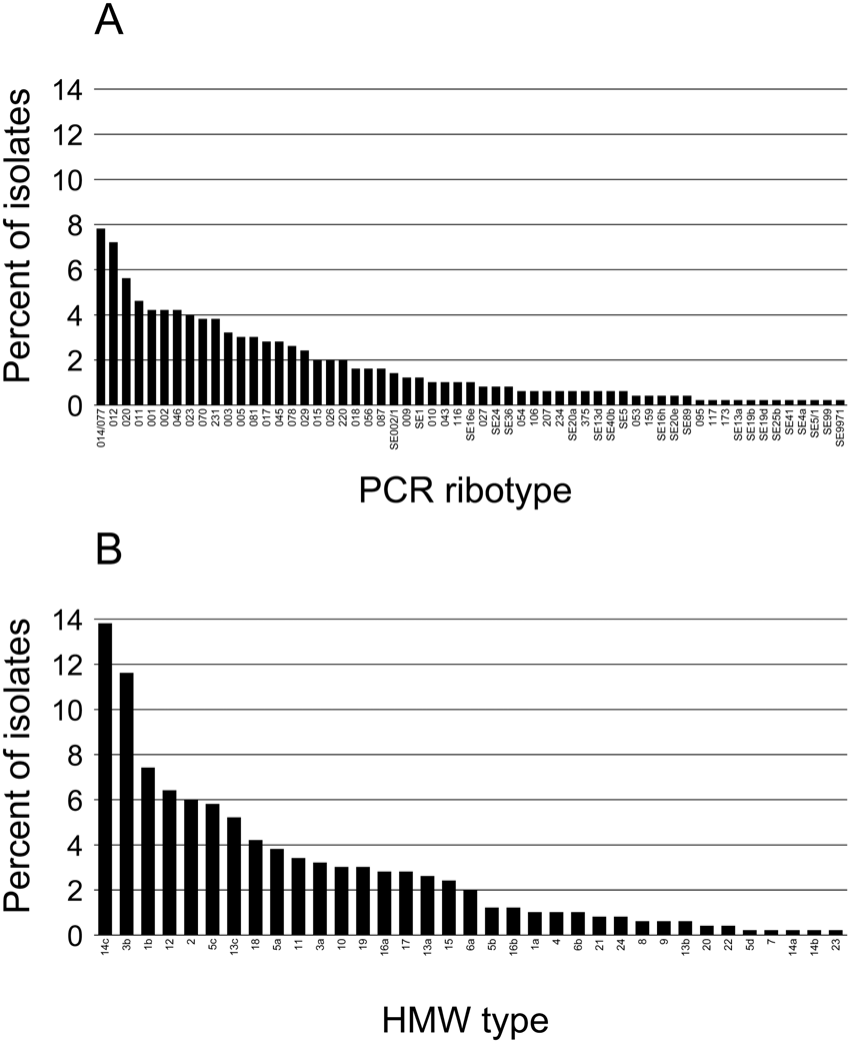
Distribution of PCR ribotypes (A) and HMW types (B) for the isolates analyzed in this study .

The initial MALDI-TOF database of 170 isolates (representing 23 PCR ribotypes) was blindly validated with a cohort of 222 isolates. The additional PCR ribotypes and HMW profiles that were detected in the validation cohort and thus missing in the initial database, were confirmed by analyzing additional clinical isolates of the same PCR ribotypes in our collection (n = 108 isolates). Among the 500 analyzed isolates a total 59 PCR ribotypes were found and 38/59 PCR ribotypes (89% of the isolates) could be grouped according to the international PCR ribotype nomenclature (Fig 1A). By comparison, the analysis by WCMS were less discriminatory and gave 24 HMW profiles (Fig 1B). Fourteen of the 24 HMW profiles (numbered as 4, 7–10, 12, 17–24) were correlated to the 13 PCR ribotypes 010, 011, 012, 015, 017, 020, 027, 046, 081, SE13a, SE13d, SE20a and SE99/1 (Table 1). Four of these 14 PCR ribotypes (011, 012, 015 and 020) could have two or more HMW profiles, e.g. PCR ribotype 012 correlated to both profile 12 and 3 (89% and 11% of the isolates, respectively).

**Table 1:** HMW-profiles and-types detected by WCMS and ETOH/FA extraction, respectively, and correlating PCR ribotypes.

HMW profile	HMW discriminatory peaks (Da)^1^	EtOH/FA extraction discriminatory peaks (Da)^2^	HMW type	PCR ribotype(s)^3^
**1**	33400 ±150, 35050 ±150-V40%, 44225 ±125	5930 (2964), lack 5958	**1a**	011 (22%)
		5958, lack 5930 (2964)	**1b**	001, 015 (20%), 087, SE4a, SE19b, SE20e, SE89
**2**	34100 ±100, 35050 ±150, 40150 ±150	N/A	**2**	002, 002/1, 159
**3**	33700 ±100, 41850 ±150	9679.6	**3a**	003
		lack 9679.6	**3b**	012 (11%), 053, 070, 117, 207, 231, SE1, SE5, SE5/1
**4**	37575 ±125, 41400 ±100	N/A	**4**	010
**5**	34100 ±200-V25%, 35050 ±150-V40%, 42450 ±150	7075 (3537)	**5a**	005, 375, SE99
		7075 (3537), 3690.5, 4099, 5776	**5b**	054, SE16h, SE19d
		7090 (3545)	**5c**	023, 116, 234, SE25b
		5019	**5d**	015 (10%)
**6**	33700 ±100, 39300 ±100	7048	**6a**	026
		7076.6	**6b**	015 (50%)
**7**	34675 ±75, 40975 ±75	N/A	**7**	SE13a
**8**	33075 ±75, 40875 ±125	N/A	**8**	SE13d
**9**	33500 ±100, 40775 ±175, 44750 ±250	N/A	**9**	SE20a
**10**	30700 ±100-V80%, 31850 ±100-V80%, 34150 ±150-V80%, 40750 ±150	N/A	**10**	011 (65%)
**11**	30700 ±100-V55%, 33275 ±125, 41325 ±125, 44625 ±225	N/A	**11**	014/077 (21%), 018, 173
**12**	34225 ±125, 39575 ±125	N/A	**12**	012 (89%)
**13**	30675 ±125-V53%, 33600 ±200, 41375 ±125	7090 (3545), 5005, 6578	**13a**	078
		7048 (3524)	**13b**	SE40b
		7076 (3538)	**13c**	029, 043, SE16e, SE36
**14**	34150 ±150, 35050 ±150, 40750 ±150	2582.5, 5820, 5960	**14a**	SE41
		lack 2582.5, lack 5820, lack 5960	**14b**	011 (4%)
		5960, lack 2582.5, lack 5820	**14c**	014/077 (79%), 020 (86%), 095, 106, 220
**15**	32275 ±75, 35050 ±150-V63%, 40750 ±150	N/A	**15**	056, SE24
**16**	32050 ±100, 40275 ±125	7091	**16a**	045
		2302, 3537, 4991, 6592	**16b**	009
**17**	33800 ±100, 39450 ±150	N/A	**17**	017
**18**	38900 ±100, 41450 ±150	N/A	**18**	046
**19**	34600 ±150, 41875 ±175	N/A	**19**	081
**20**	34800 ±100, 40250 ±50	N/A	**20**	015 (20%)
**21**	33300 ±100, 35050 ±150, 41350 ±150	N/A	**21**	020 (14%)
**22**	34150 ±150, 35050 ±150, 41400 ±100	N/A	**22**	011 (9%)
**23**	38350 ±50, 41375 ±100	N/A	**23**	SE99/1
**24**	30700 ±100-V50%, 33900 ±100, 44250 ±150	N/A	**24**	027

^1^ The average and the ± variation of HMW-peaks for each profile are shown in Da. A peak that was not detected in 100% of the spectra is labeled by "V" for variable and the incidence is shown in %.

^2^EtOH/FA discriminatory peaks were present or absent in 100% of the spectra analyzed. Ions in brackets depict the mass of doubly charged ions.

^3^Include the types (n): 001 (21), 002 (21), 002/1 (7), 003 (16), 005 (15), 009 (6), 010 (5), 011 (23), 012 (36), 014/077 (39), 015 (10), 017 (14), 018 (8), 020 (28), 023 (20), 026 (10), 027 (4), 029 (12), 043 (5), 045 (14), 046 (21), 053 (2), 054 (3), 056 (8), 070 (19), 078 (13), 081 (15), 087 (8), 095 (1), 106 (3), 116 (5), 117 (1), 159 (2), 173 (1), 207 (3), 220 (10), 231 (19), 234 (3), 375 (3), SE1 (6), SE4a (1), SE5 (3), SE5/1 (1), SE13a (1), SE13d (3), SE16e (5), SE16h (2), SE19b (1), SE19d (1), SE20a (3), SE20e (2), SE24 (4), SE25b (1), SE36 (4), SE40b (3), SE41 (1), SE89 (2), SE99 (1), SE99/1 (1)

The remaining 10/24 HMW profiles corresponded to multiple PCR ribotypes and a second MALDI-TOF analysis using EtOH/FA extraction was performed in an attempt to improve the resolution. The combination of WCMS and EtOH/FA extraction (collectively called HMW typing) increased the number of discernable types from 24 to 35, and additional PCR ribotypes, e.g. 003, 009, 026, 045, 078, SE40b and SE41 could be resolved (Table 1). The biomarker peaks could also be detected by the less time consuming, direct transfer of bacterial cells to the MALDI target plate overlaid with 1 μl FA prior to HCCA matrix application, but a full EtOH/FA extraction should be adopted for best resolution of the biomarker peaks (data not shown).

Including isolates with known international PCR ribotype nomenclature only (n = 38 types) there was a 52% probability for a HMW type to correlate to a PCR ribotype, and vice versa, and 82% probability for a PCR ribotype to correlate to a HMW type (Table 2). There were no false positive or negative found in the HMW type validation cohort (222 strains) when compared to the derivation cohort.

**Table 2:** Simpson’s index of diversity and Wallace coefficients of PCR ribotyping and HMW typing.

Method	No. of partitions^1^	Simpson’s ID (CI 95%)	Adjusted Wallace (CI 95%)	
			HMW typing	PCR ribotyping
PCR ribotyping	38	0.960 (0.955–0.964)	0.847 (0.801–0.892)	
HMW typing	29	0.936 (0.928–0.945)		0.523 (0.488–0.557)

^1^Only isolates with known international PCR ribotype nomenclature was included in the analysis (n = 443). Values were calculated using the online tool at http://darwin.phyloviz.net/ComparingPartitions/index.php?link=Home

The method developed in this study, HMW typing, is in our mind an important complement to other typing methods, specifically for screening purposes in hospitals, environment, or in animal settings. The national surveillance program in Sweden includes PCR ribotyping covering 4% of CDI patients per year. Although this surveillance strategy has been able to detect geographical clustering and local outbreaks of the PCR ribotypes 012, 017 and 046 [10], the number of typed strains is too small to investigate the extent of such local outbreaks. As MALDI-TOF has become a standard tool in many clinical laboratories, the HMW typing method has the potential to rapidly analyze a large number of strains and improve local surveillance at a low running cost. In fact, HMW typing was used to monitor the first outbreak of moxifloxacin-resistant *C*. *difficile* PCR ribotype 027 in Sweden (in the county of Kronoberg, January 2014). The clinical laboratory in Kronoberg adopted HMW typing to follow the outbreak, and all HMW type 24 isolates were later confirmed as PCR ribotype 027 (personal communication Åsa Johansson, Växjö). A concern that this strain had spread to other hospitals was raised, and the Public Health Agency of Sweden immediately initiated screening of moxifloxacin-resistant *C*. *difficile* isolates in Sweden using HMW typing. During a two-month period, 182 isolates from 17 laboratories were typed and only 2/182 moxifloxacin-resistant isolates of PCR ribotype 027 were detected. Thus, it was rapidly concluded that the outbreak was geographically limited to the county of Kronoberg. A benefit of the method is the short turnaround time, enabling hospitals, laboratories and infection control specialists to react within a short notice on any signs of spread. Having visible colonies on an agar plate, it takes approximately 15 minutes to determine the HMW type (and 30 minutes if EtOH/FA extraction is also performed) i.e. considerably faster than PCR ribotyping. Last but not least, the reagent cost for HMW typing is very low—less than 0.5$ per sample. One limitation of this study is that the majority of isolates were from one country only, and there may be different results in other countries and parts of the world due to local strain variation. However, the strains from the Cardiff-ECDC collection are derived from other countries and the clinical isolates shared the same PCR ribotype as well as HMW type with these reference strains. We thus believe that the method is stable and should be useful also for international comparisons.

Other benefits than speed and costs may also be had. Since several HMW types could be found for certain PCR ribotypes, consistent with the study of Dingle *et al*. 2013 (12), combining HMW typing and PCR ribotyping results may improve epidemiological analysis. As HMW protein profiles most likely are dominated by the *C*. *difficile* S-layer proteins amongst other large molecular weight proteins [7], the method could also give information regarding virulence. SLPs from *C*. *difficile* have been shown to be involved in binding to gastrointestinal tissues and to induce inflammation [13–15]. Furthermore, SLPs could be involved in the immunological response and, together with antibodies against the *C*. *difficile* toxins, aid in the protection against disease or relapse [16]. HMW-typing enables monitoring of SLP mutations or switching in various PCR ribotypes over time [12], allowing for a parallel analysis of their role in virulence, immunity and disease severity.

## Supporting Information

S1 Fig Comparison of spectra from SLP extractions and WCMS of PCR ribotype 003/HMW profile 3 (A) and of PCR ribotype 015/HMW profile 1 (B). The upper, middle and lower panels for each of the types shows acidic glycine extraction of SLPs, urea extraction of SLPs and WCMS analysis, respectively.
(TIF)
