# Supplementary figures and images for: High Molecular Weight Typing with MALDI-TOF MS - A Novel Method for Rapid Typing of Clostridium difficile
Source: PLoS One. 2015 Apr 29;10(4):e0122457. doi: 10.1371/journal.pone.0122457 (PMC4414534; doi:10.1371/journal.pone.0122457)

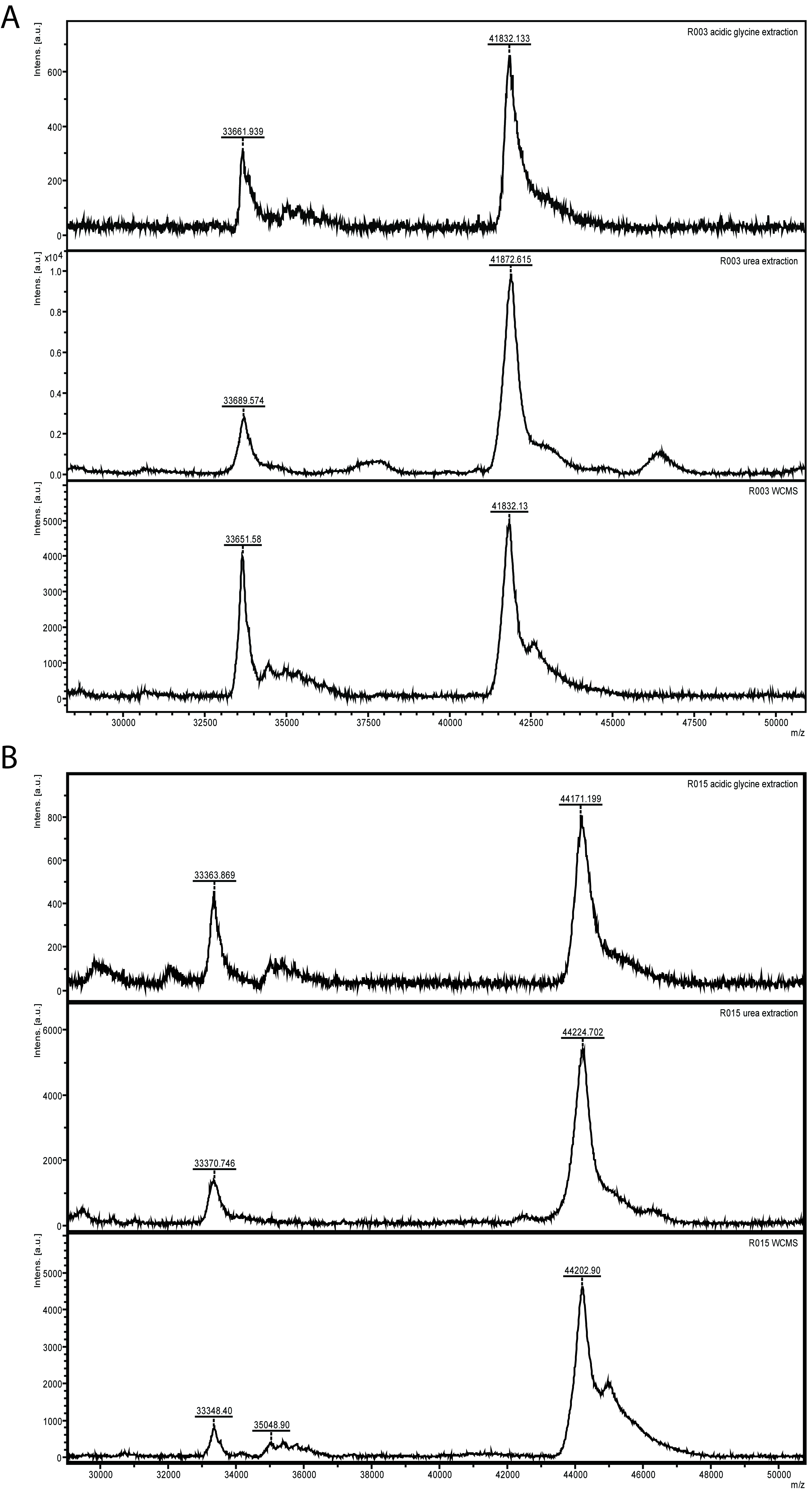

Supplement: S1 Fig — The upper, middle and lower panels for each of the types shows acidic glycine extraction of SLPs, urea extraction of SLPs and WCMS analysis, respectively. (TIF) [file pone.0122457.s001.tif]
